# Commentary: Acceptable performance standards for clinical laboratory methods

**DOI:** 10.1155/S1463924682000029

**Published:** 1982

**Authors:** Callum G. Fraser

**Affiliations:** Department of Clinical Biochemistry Flinders Medical Centre Bedford Park South Australia 5042 Australia


Journal of Automatic Chemistry, Volume 4, Number 1 (January–March 1982), pages 1–2

### Editorial

### 1982: A new start for the Journal

Many of you will have noticed in the fourth issue of Volume 3 that the *Journal* has recently been acquired by Taylor & Francis Ltd. This will add the considerable experience of a scientific publisher, founded in 1798 with over 30 journals already in their portfolio, to the active editorial team established by myself and United Trade Press. During its first three years, *Journal of Automatic Chemistry* has grown at a steady rate, both in terms of content and subscription and advertising income. I am grateful to United Trade Press for their support in founding the *Journal*.

Taylor & Francis Ltd, together with the existing Editorial Board, aim to increase the subscription level by introducing the *Journal* to a wider audience. The content will continue to be vigorously refereed, although the turn round time from receipt of paper to publication will be improved. The size of the *Journal* and its frequency will also be reviewed over the ensuing year. In order to cater for the real needs of the readership, it is important for you, the reader, to let us know either directly or through members of the Corresponding Editorial Board your views of the *Journal*. Are there areas that you would like covered, but at present are not being addressed?

### What has the Journal achieved?

In its short life, the *Journal* has achieved some success and status—recently in *Nature* (October 1981) Professor T. S. West wrote:

> Each issue contains about eight or nine articles, many of them devoted to microprocessors and a smaller number to improvements to commercial automated systems or the construction of miscellaneous ancillary devices to improve their performance. In addition there are useful and informative articles on meetings, new products and literature, and a running calendar of current and forthcoming events. There are also occasional book reviews.
>
> The standard of the papers is usually good, even excellent, and the 'virtual' A4 format allows good use to be made of illustrations. The generally high quality of the articles read in the sample issues suggests that this journal has so far done an excellent job and should be scanned on a regular basis by all who are concerned with automation. It is not possible, since the dates are not given, to establish publication times, but the journal gives the impression of being very much 'on the ball'.

This shows that the aims and objectives the Editorial Board set out to achieve when *Journal of Automatic Chemistry* was launched have been met. However, in such a changing area it is important to keep abreast of the technology. This we will endeavour to do by submitted articles, by evocative and informative commentaries, both from Editorial Board members and from invited specialists, and by review articles. The enthusiasm and experience of our new publisher will be a considerable asset as the *Journal* develops.

**Peter B. Stockwell**

### Commentary

### Acceptable performance standards for clinical laboratory methods

Many of the instruments and methods used in clinical laboratories have been selected for somewhat subjective reasons, such as low cost and ease of performance, rather than as a result of objective evaluation of their analytical performance. The usual clinical laboratory quality-control procedures monitor performance characteristics such as imprecision and inaccuracy in order to detect changes in performance, but they cannot improve an analytical instrument or method which is basically unsound [1]. Therefore, an essential part and first step of a *total* laboratory quality-control programme should be the evaluation of analytical instruments or methods before their introduction into the routine diagnostic service laboratory.

New analytical instruments and reagent kits are introduced each year. Evaluation of their performance characteristics is a complex procedure which requires considerable expertise, skilled staff, ample space and time resources, and suitable patient samples and comparative methods. There are many published protocols for evaluation; these have been recently discussed in detail by Westgard [2]. Although no protocol is universally applicable, most protocols proposed in the literature follow a similar pattern. The majority of published evaluations conform, in general experimental design, to this pattern. Therefore, evaluations cannot be performed by *all* potential purchasers and many must rely for guidance on objective reports in the literature, or the experience of professional colleagues.

A major problem with published evaluation protocols is that definitive criteria for acceptability of the performance characteristics are not delineated. Indeed, many evaluations of instruments, reagent kits, and methods document in great detail all aspects of analytical performance, but fail to assess in an objective manner whether the performance found is truly suitable for clinical laboratory use.

It has been stated that one of the major current philosophical problems in clinical biochemistry is the assessment of the standard of analytical performance that is actually required to provide optimal patient care at least expense [3]. Such standards have been termed *analytical goals*. One of the major difficulties in the setting of such goals is that clinical biochemistry tests are used in many different clinical settings, such as in aiding diagnosis, in screening, in assessment of the efficacy of therapy and in emergency situations. This has led some clinical biochemists to consider the definition of numerical analytical goals to be an insoluble problem; for example, the Expert Panel on Nomenclature and Principles of Quality Control of the International Federation of Clinical Chemistry state that a single set of performance characteristics is unlikely to be applicable to all of the situations in which tests are used [1]. However, analytical goals for a number of performance characteristics have been documented in the literature; this subject has been recently reviewed [4].

Most work has been concerned with the delineation of analytical goals for imprecision; this is considered to be appropriate since analytical imprecision cannot be avoided. Strategies for the derivation of goals for imprecision have been classified as being based on (1) the reference range; (2) biological variation; (3) the views of clinicians; (4) the state of the art; (5) the

0142–0453/82/0401 0001 $05·00 © 1982 Taylor & Francis Ltd consensus opinions of expert groups; and (6) the views of individuals. The recommendations made by Barnett [5], which are based upon a synthesis of opinion of clinical and laboratory specialists, are probably the most widely adopted. However, the current view would be that goals for the imprecision of plasma analyses are best derived from intra-individual biological variation. If biological variation data are unavailable, then goals should be derived from the state of the art achieved by a selected group of better laboratories. Goals based upon biological variation have been recently summarized by the Subcommittee on Analytical Goals in Clinical Chemistry of the World Association of Societies of Pathology [6] and summaries of the current performance attained by better laboratories have been documented by Stevens and Cresswell [7].

There has been little work performed on setting analytical goals for the imprecision of analyses of biological fluids other than plasma or serum. Goals for the analysis of common urine constituents have been derived from biological variation, the state of the art and surveys of clinical opinion [8]. It appears that the biological variation strategy is not totally applicable for this biological fluid and it has been suggested that, since all the current strategies adopted for the delineation of goals do have disadvantages, the most stringent goals obtained should be applied.

Goals for inaccuracy have been delineated by few authors. It is generally believed that, at the present time, clinical laboratories should be attempting to eliminate bias from their results in order to make results as far as possible comparable over time and geography. Therefore, the goal for inaccuracy is that methods should have no bias.

Imprecision is usually defined to mean random analytical error, while systematic error is called inaccuracy or bias. However, bias can be considered to have two components: systematic bias, which reflects the difference between results found and their true values; and random bias, which reflects non-constant changes in inaccuracy brought about by, for example, changing the vials of a calibrator for every analytical batch. Thus, it is difficult to fully separate inaccuracy from imprecision. It may therefore be of advantage to consider the goals promulgated as goals for imprecision as goals for *total analytical error*. The total error concept [9] also facilitates communication with clinical staff and manufacturers; it is hoped that this concept will become much more widely used by clinical biochemists.

A thorough evaluation also investigates performance characteristics such as detection limit and turnaround time. Goals for these characteristics should also be used, wherever possible, for objective analysis of experimental data. There are, however, few publications concerning facets of the discipline such as these. It is hoped that work in these and further areas will be performed in the near future.

The derivation of analytical goals for all facets of clinical laboratory analyses is itself a fascinating subject, but it is the sound application of goals that will ultimately benefit patient care. Goals should be used in individual laboratories in order to assess their quality-control results; comparison of found performance with analytical goals should allow laboratories to pick out those methods which require improvement. Goals should also be used in the setting of acceptable standards of performance by accreditation or other regulatory agencies. Laboratories could use a comparison of their performance with published goals as an additional lever to obtain new analytical equipment. Published goals could aid laboratories in communication with clinicians if allegations that a particular laboratory test was inadequate were to be refuted in an objective manner, provided that the goals were met. Manufacturers of analytical instruments and reagent kits have played a notable role in the development of clinical biochemistry and it could be argued that the present analytical state of the art has been, in large part, set by commercial interests; it is believed that analytical goals, set by the profession, should be taken very much into account when manufacturers develop new instruments or kits and when laboratories are considering the purchase of new analytical techniques. Finally, it is surely no longer satisfactory for published evaluations of instruments and reagent kits to merely use professional judgement or the specifications of the manufacturer as criteria of acceptability; it is firmly believed that it should be *mandatory* for all evaluators to carefully compare the performance obtained with analytical goals and to make objective judgement on the acceptability, or otherwise, of as many of the performance characteristics as possible.

### References

1. Buttner, J., Borth, R., Boutwell, J. H., Broughton, P. M. G. and Bowyer, R. C., *Clinica Chimica Acta*, **98** (1979), 145F.
2. Westgard, J. O., *CRC Critical Reviews in Clinical Laboratory Sciences*, **13** (1981), 283.
3. Barrett, A. E., Cameron, S. J., Fraser, C. G., Penberthy, L. A. and Shand, K. L., *Journal of Clinical Pathology*, **32** (1979), 893.
4. Fraser, C. G. in *Progress in Clinical Pathology*, Vol. 8. Ed. Stefanini, M. and Benson, E.S. (Grune and Stratton, New York, 1981), p. 101.
5. Barnett, R. N., *American Journal of Clinical Pathology*, **50** (1968), 671.
6. Proceedings of the Subcommittee on Analytical Goals in Clinical Chemistry, WASP, *American Journal of Clinical Pathology*, **71** (1979), 624.
7. *NewsSheet* (Association of Clinical Biochemists Ltd, 1979), No. 197, 14.
8. Shephard, M. D. S., Penberthy, L. A. and Fraser, C. G., *Clinical Chemistry*, **27** (1981), in press.
9. Westgard, J. O., Carey, R. N. and Wold, S., *Clinical Chemistry*, **20** (1974), 825.

**Callum G. Fraser**
*Department of Clinical Biochemistry, Flinders Medical Centre, Bedford Park, South Australia 5042, Australia*

